# Social networks and customer loyalty: review of loyalty keys and main social networks publications’ characteristics

**DOI:** 10.3389/fpsyg.2023.1286445

**Published:** 2023-10-20

**Authors:** Nélida Dávila Espuela, Maria Dolores Reina Paz, Claudia Sevilla Sevilla

**Affiliations:** Department of Business Economics and Accounting, Universidad Nacional de Educación a Distancia (UNED), Madrid, Spain

**Keywords:** loyalty, social network, social network characteristics, loyalty model, customer loyalty, brand

## Abstract

The aim of this work is to shed light on the characteristics and relationship between customer loyalty and publications by the companies on social networks; it has been undertaken with the aid of an exhaustive review of previous studies from 1994 up to the present time. The purpose of the research is to generate a model that can tackle the practical characteristics of the publications on social networks to encourage loyalty. With a view to this, a model is developed that is an extension of the traditional “Four Stage Loyalty Model” based on other constructions of the same model, and combined with the characteristics of social networks publications defined in earlier literature. This reflexive approach is particularly important here due to the fact that companies have to be closer to customers’ requirements and customers have the option to choose from which type of communication they wish to be the object.

## 1. Introduction

According to Jang et al. (2008), working on the digital communities of brands, firms can achieve the loyalty of their customers. Consumers generally follow brands that represent their image and personality, which is why the interaction that is generated in brand communities that emerge in an online environment are very effective when it comes to retaining customers with different profiles. According to the study by Van Asperen et al. (2018) social network consumption alone, which can be considered a passive engagement, is now directly associated with loyalty.

Grace et al. (2020) on following a brand on social networks, found that the consumers generate a loyalty bond that takes in different dimensions. It is not just a commercial bond, but an emotional link is established through the social networks, together with a proactive relationship with the brand.

As has been established in previous studies, loyalty implies, amongst other competitive advantages, that the customers are less sensitive to prices and, so, they stick to the brand for longer without taking any notice of the competition and its potential offers. This means that brand loyalty is also very useful for preventing customers from changing to the competition, given that the more loyal customers are, the less vulnerable they will be to the competitions’ activities.

Furthermore, capturing new customers amounts to a financial cost and an investment in time that is up to six times greater than keeping existing customers loyal, according to Hallowell (1996). And, not only is capturing customers more costly than holding on to them, but it is also true to say that loyal customers amounts to less investment in time and attention than new customers, which means profit with less expenses for the company. Moreover, throughout the active life of a purchaser, customers who are loyal to a brand, can prove to be worth up to 10 times more than “disloyal” customers. Not only because of the long period that they remain as customers but also because of the lower amount the firm spends on retaining them (Anderson and Srinivasan, 2003).

Another direct benefit for companies, which also leads to a cost reduction, is the fact that it provides the company with more information about consumer behavior. The awareness and knowledge of loyal customers can be used to improve the effectiveness of marketing activities and negotiations with the customers (Russell-Bennett, 2001).

At present, most companies have understood that the service to the customer is essential, which means that this is no longer a differentiating action, so it is necessary to seek new ways of differentiation, such as focusing on the customer’s experience (Brun et al., 2017). It must be borne in mind that with the digitalization of the relationships between customers and companies, the consumers’ experience goes far beyond logical thought and the traditional purchase decision process, incorporating an emotional aspect that is just as valuable as the aspects that were valued until the present time (Gentile et al., 2007).

Firms find social networks to be a channel for reactivating their branding, listening, segmenting, talking, conversing, mobilizing, helping and involving potential customers to turn them into loyal users. In view of the major competition, current businesses have found themselves obliged to seek new social networks strategies for their loyalty programmes in order to attract and keep customers loyal so they can continue to be competitive (He et al., 2019).

The aim of the brands where social networks are concerned has to be much more than merely being present, given that this relationship with its customers may offer them many more advantages, such as interacting with loyal customers, having an influence on the members of a community and their perceptions about the brand, disclose information and teach the customers, affecting their opinions and needs (Algesheimer et al., 2005).

This new environment means the need for a major reconversion of business strategies. In the market, changes take place in the way that one communicates, collaborates, consumes, and creates content and value. Nguyen et al. (2015) introduce the concept of the strategic capacities of social networks or the capacities firms have to integrate their knowledge acquired of the skills in using social networks in the company’s strategic direction.

Digital marketing involves improvements in efficiency when compared to traditional marketing and adds the option of immediate response from the customers, this implies interesting advantages on multiple levels. Companies’ success currently depends, and increasingly so, on their presence on social networks and the creation of interesting and interactive communication, as well as the presentation of offers to the intern3.auts who are spending increasingly long periods of time on social networks to interact with other people, keeping up to date with the latest news, commenting on events and situations and sharing the posts of others (Dabija et al., 2018).

Several factors have been studied that affect brand loyalty on social networks, however, there are no studies that detect which specific characteristics of the publications on social networks have the greatest effect on consumers, which is therefore the main objective of this research.

## 2. Methodology

A review has been carried out of the empirical studies published so far concerning two subjects: loyalty and social networks. This review of past literature is carried out from 2020 until 2022, priority being given to the works published by Web of Sciences. The study methodology was based upon the principles of Bramer et al. (2018), a systematic search for relevant studies being established as from a research question, which led to a synthesis that gave rise to a new model that incorporated recommendations to be put into practice.

The following keywords associated with the subject matter of the study were used in order to access the greatest number of papers possible, and those words were utilized both individually and in association, when looking for the topic: loyalty, social media, and social networks. A large number of articles turned up as a result of this search, so it was necessary to filter them by content.

With the keyword loyalty, a large volume of bibliography referring to fidelity programs was excluded, as they are not the subject of the study, together with specific countries and sectors. Papers are included that refer to how the term has evolved, definition, advantages, and the practical use of the term.

With social networks, studies concerned with the use of one network in particular were excluded, because this intends to be generic. The subject matter from specific sectors and countries was also excluded. However, papers about the emergence of social networks, the way they have evolved, their characteristics and benefits to companies were included.

**FIGURE 1:**
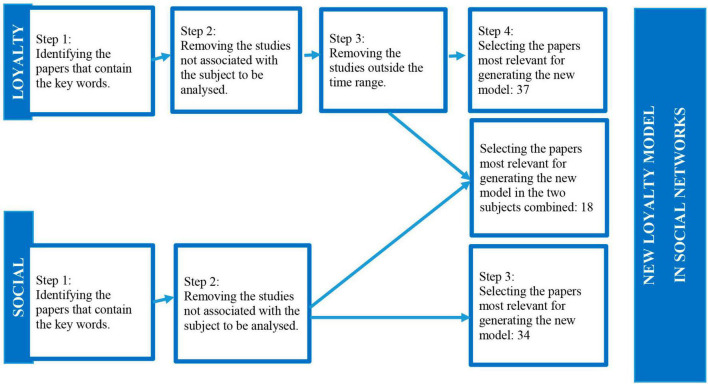
Methodological approach to reviewing the literature. Source: Own research.

Once the loyalty model that constituted the foundation for subsequent ones had been identified, (Oliver, 1997), it was established that the years prior to the generation of this model (1997) would be the first to be taken into account in the study, in order to be able to understand its origins. Thus, 1994 was the year as from which research took place, because that was the year in which Dick and Basu (1994) developed the model, which is clearly the precursor of the model by Oliver (1997), and clearly enables us to comprehend its development. Methodological approach can be seen in Figure 1.

In the case of social networks, as it is a relatively recent subject, no explicit time segmentation is carried out for the previous studies, the segmentation merely being limited to the content itself.

The articles that were found as a result of the research and filters described were used to define the background to the loyalty and social networks criteria. Moreover, the most representative models were identified and used to generate our own model with practical solutions.

## 3. Conceptual framework

### 3.1. Loyalty background

Companies’ success can be measured in terms of the loyalty of their customers (Nyadzayo and Khajehzadeh, 2016). Brand loyalty reflects the extent to which customers are interested in that brand (Aaker, 1997). This concept is so important that market researchers have been focusing on it for more than 60 years (Costa Filho, 2019).

Obviously, brand loyalty can only exist if customers have different brands to choose from, so there can be no such thing as brand loyalty in monopoly situations (Mellens et al., 1996). A long-lasting and stable relationship between brands and customers can only be forged if the former can understand the needs of the latter, and this has to be one of the main concerns in marketing.

Customer loyalty was traditionally interpreted as meaning repurchasing the same brand. However, after numerous acts of research, a new tendency emerged that attributed a customer attitude component to loyalty. Admitting that repurchasing the product or service is an important aspect of customer loyalty, Mellens et al. (1996) argue that it cannot be the only factor for rating this characteristic that is so important to a company’s customers. Repeating a purchase is not necessarily the same as proof of loyalty in itself, it could be due to consumer inertia, because they simply cannot be bothered to spend time on seeking alternatives.

**FIGURE 2:**
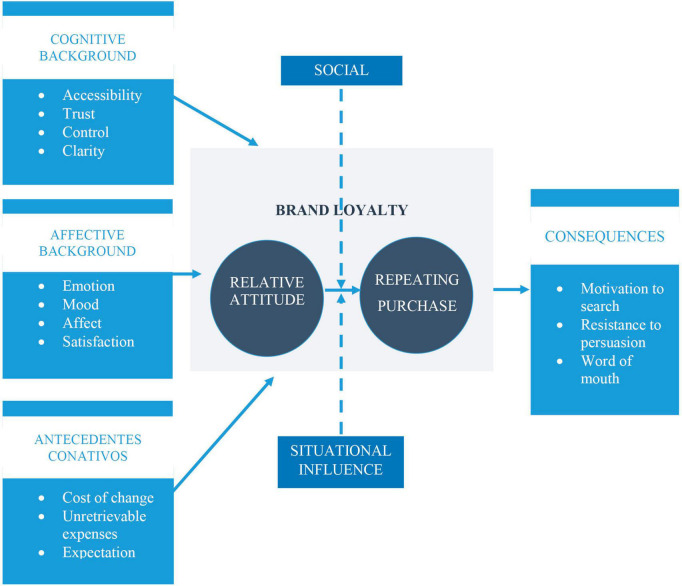
Dick and Basu’s Loyalty model. Source: adapted from Dick and Basu (1994).

According to this trend, a distinction should be made between genuine loyalty and “spurious loyalty” or false loyalty, which merely involves buying the product again. It could be explained by contextual factors such as low prices, better exposure of the product or simply by the absence of other options at the moment of purchase (Dick and Basu, 1994). These authors find that loyalty clearly has an attitudinal component that they show in the following way in Figure 2.

**FIGURE 3:**
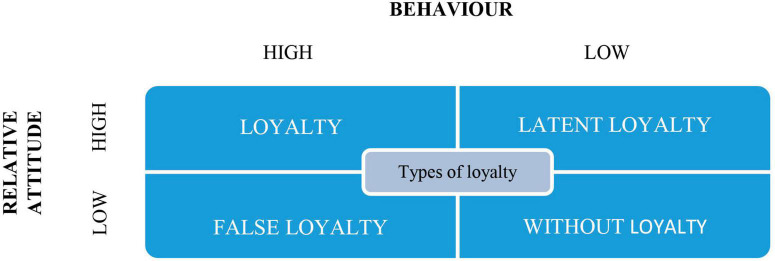
Dick and Basu’s Loyalty typologies. Source: adapted from Dick and Basu (1994).

On the basis of this dual perspective (behavioral and attitudinal), the authors prepared a table of customer loyalty typologies in Figure 3.

Mellens et al. (1996) made the same comparison between behavioral and attitudinal when defining brand loyalty as can be observed in Table 1.

**TABLE 1:** The behavioral and attitudinal measures of loyalty by Mellens, Dekimpe and Steenkamp.

	Advantages	Disadvantages
Behavioral measures	Based on current behavior patterns Not incidental Easy to recall	Repetition of purchase is not distinguished from genuine brand loyalty More sensitive to short-term fluctuations Difficult for making correct decisions
Attitudinal measures	Repetition of purchase separate from brand loyalty Less sensitive to short-term fluctuations Better for correct decision-making	Valid representation of reality not guaranteed Incidental More difficult to recall

Source: adapted from Mellens et al. (1996).

The Dick and Basu Model is a sound base for the highly studied model defended by Oliver (1997). Oliver’s Model, brings together in a simplified way, the concepts of loyalty by other authors, as is the case with Grace et al. (2020), who state that after passing through a process of assessment of the brand stimulants (its attributes and benefits), cognitive assessment (satisfaction), behavioral (repetition of purchase) and emotional (commitment to the brand), a brand loyalty relationship is eventually reached that is characterized by a relationship with the customer consisting of: cognitive interdependence, removal of alternatives, positive expectations, pardoning possible errors and willingness to sacrifice.

Oliver (1997) defines customer loyalty as a commitment to repurchase that is kept in the future despite the marketing efforts made by other brands. But not only that. It introduces the Four Stage Model. A simple model that includes the four stages of loyalty:

• Cognitive loyalty: at this phase, loyalty is determined by the information that the consumer receives.
• Affective loyalty: is the consumer’s attitude toward the brand.
• Conative loyalty: implies the wish to carry out an action.
• Action loyalty: is the effective action, normally the purchase.

**FIGURE 4:**
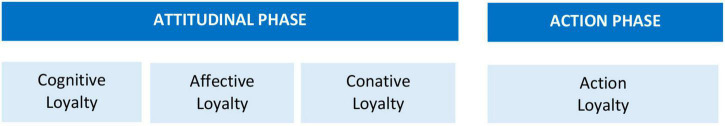
Oliver’s Loyalty model. Source: adapted from Oliver (1997).

This four-stage loyalty model implies that the different aspects of loyalty do not emerge simultaneously, but consecutively. Numerous models will be based on this one in the future, because it encapsulates and simplifies most of both the past and future definitions in Figure 4.

The same author, in Oliver (1999) defined brand loyalty as a great commitment made by the customer to repurchase the product or service or to constantly recommend a product or service in spite of the marketing activities of another company to capture that person as a customer. As can be seen, this definition contains other concepts in addition to merely purchasing or repurchasing the product, including attitudinal aspects that make customers fail to react when faced with other potential actions taken by the competition to attract a change in their purchasing activity.

**FIGURE 5:**
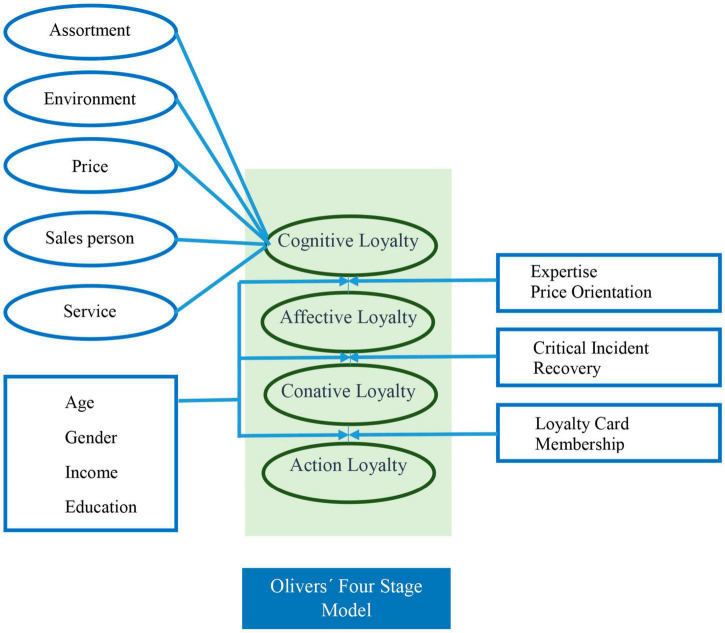
Evanschitzky and Wunderlich’s loyalty model. Source: adapted with permission from Evanschitzky and Wunderlich (2006).

Several authors base their studies on Oliver’s loyalty model creating other models from Oliver’s. This is the case with Evanschitzky and Wunderlich (2006), who incorporate moderating effects into Oliver’s model, as can be observed in Figure 5.

**FIGURE 6:**
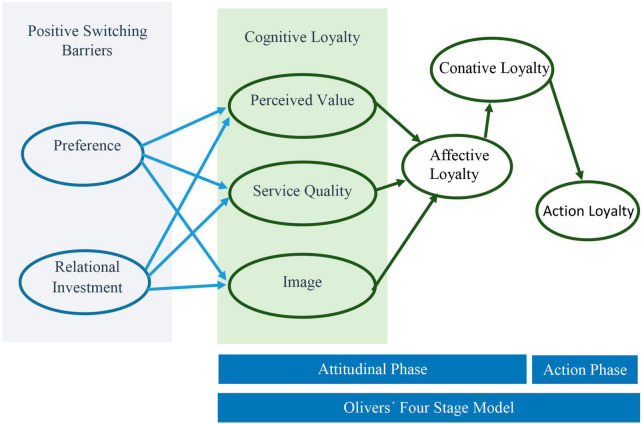
Han and Hyun’s Loyalty model. Source: adapted with permission from Han and Hyun (2012).

Another example of a model based on the Four Stage by Oliver is Han and Hyun (2012) that, keeping the foundations of the 4-stage model virtually intact, including the positive entry barriers: The adaptation of this model is shown in Figure 6.

**FIGURE 7:**
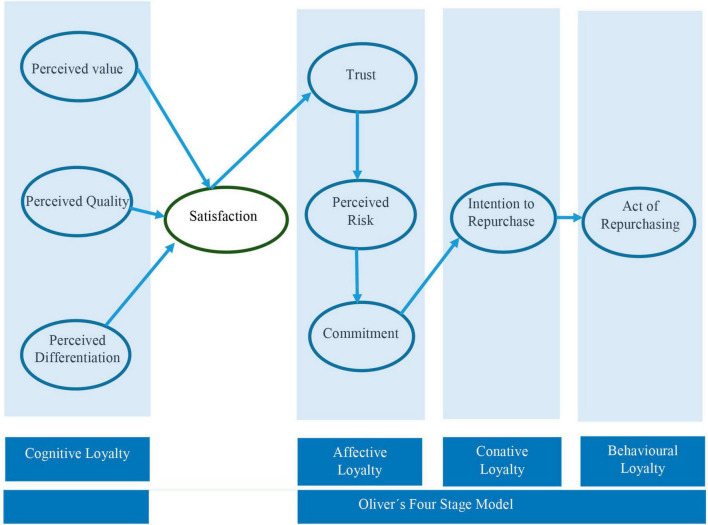
Loyalty model by Costa Filho (2021). Source: adapted from Costa Filho (2021).

In the case of Costa Filho (2021) the concept of satisfaction is included as a step prior to affective loyalty as can be observed in Figure 7.

As can be observed, Oliver’s Four Stage Model has been used as the basis for developing different models such as those shown above thanks to the combination of their multi-dimensional simplicity expressed by other authors. That is why it will be utilized as the basis for the model presented in this study.

### 3.2. Social networks background

According to Chu and Kim (2011), social networks are a space that came into existence as a result of the need to express oneself and communicate, which could become just another channel for conversation and connection with acquaintances, friends, and relatives and that is used by individuals from different generations. At present, there are 4.59 billion users of social networks, and it is expected that there will be a progressive growth to 5.85 billion users by 2027 (Williams and Cothrel, 2000).

There are two types of social networks according to Burgueño (2009):

• Horizontal social networks: with users of different profiles and a variety of interests. They do not focus on one particular topic. For example, Facebook.
• Vertical social networks: with one specific and common objective for all the users. For example LinkedIn.

Research work dating back more than 10 years, already indicated that social networks would be the ideal tool for orientating the customer (Castello-Martinez, 2009). Manchanda et al. (2015) stated that the communities of online customers (such as the sites of social network firms), could provide companies with considerable economic profit, increasing customers’ expenditure.

Kaplan and Haenlein (2010) found that social networks were services within websites that enabled users to:

• construct a public or semi-public profile within a limited system,
• articulate a list of other users with whom to share a connection and,
• display and track a list of contacts and those prepared by other users within the environment of the social network.

Nowadays, social networks have become a daily ritual for the majority of their users (Krishen et al., 2016).

**FIGURE 8:**
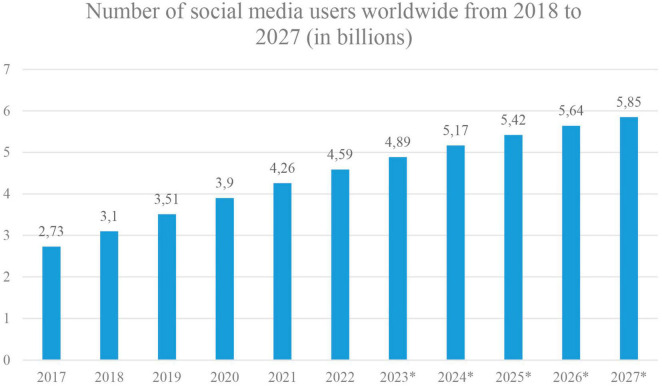
Evolution in the use of social networks in the world. Source: adapted from Statista (2022).

The emergence of different social networks, the socialization of access to the new technologies and the diffusion of these networks, have ensured that the increase in the use of social networks has been constant. Figure 8 adapted from Statista (2022) shows this steady growth since 2017 and the growth that is expected to take place in the coming years.

As can be seen, the use of social networks has clearly and increasingly been making major inroads.

In the USA in 2022 the utilization of social networks had reached 90%. A total of 55% of the users are women (Statista, 2022).

Different social network platforms have been appearing throughout the years, adapting to the users’ preferences and needs. Some of them are more focused on information, such as Twitter, whereas others offer mixture of information and images, such as Facebook or Instagram, and others, like YouTube and Pinterest, are more orientated to images or videos. At the present time, the most extensively used social network is Facebook (Statista, 2022) with 2.9 million active users in the world, followed by YouTube with 2.5 million, WhatsApp with 2 million, and Instagram with 1.5 million.

According to Williams and Cothrel (2000), social networks are a brilliant business strategy that offers a great opportunity for branding. Consumers are turning into influence references of the brand images with which they interact (Cova and Dalli, 2009) because the interactions between brand and consumer have a major impact on the companies. Therefore, social media plays a significant role in customer loyalty (Elareshi et al., 2023).

## 4. Justification and proposal for the model

**FIGURE 9:**
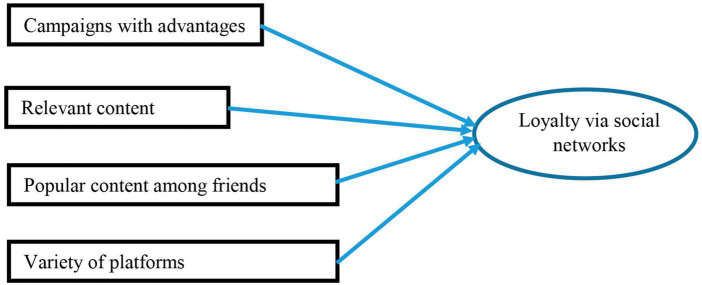
Model for loyalty on social networks by Erdogmus and Cice. Source: own research based on Erdogmus and Cicek (2012).

The work by Erdogmus and Cicek (2012) proposes a generalist model, without specifying any specific sector, for the impact of marketing on social networks where customer loyalty is concerned, as can be observed in Figure 9.

However, this model was not referenced or extended subsequently in other studies. Erdogmus and Çiçek’s model does not develop the loyalty dimension taking into account the different variables that make up the loyalty concept. Therefore, there is a gap in the current literature in this sense regarding a comparison between models that feature a combination of the two concepts.

The model proposed in this work has two dimensions: customer loyalty and the characteristics that are most important in the publications in social networks to achieve customer loyalty.

### 4.1. Social networks

It is very easy for a large proportion of the publications made by companies to be ignored by social network users. If there is no steady flow of popular and interesting publications, it is highly likely that users will lose interest in the websites (Banerjee and Chua, 2019).

This means that it is essential for the brand presence on social networks to be detectable, connected, opportune and perceptive (Pinto and Yagnik, 2017) so that it can attract the followers’ attention and interest. Furthermore, social media marketing positively impacts customer loyalty (Sohaib and Han, 2023).

#### 4.1.1. Relevant content

The first characteristic of brand communications on the social networks will be that the content of those publications is interesting and useful to the users who follow them, otherwise those followers will lose interest. Users deliberately select specific media and in the case of social networks, specific pages where there is content that satisfies their needs (Zhang et al., 2011).

Companies must inform themselves well about the type of content that interests their consumers and offer them it; this would be the way to get them interested and get them hooked on this brand on the social networks. To successfully achieve this aim, it is necessary to listen to the customers, understand them and offer them that interesting content that they are waiting for Krishen et al. (2016).

Products associated with the brand and interesting content generated by the brand itself can be accessed via the social networks, while at the same time the users are feeling the benefits of the consumers’ knowledge and experience (Shu and Chuang, 2011). Achieving these targets will mainly be what makes a content relevant.

One of the biggest advantages of social networks is that they can reach customers and offer them this relevant content without interrupting their browsing, in contrast to traditional advertising (Castello-Martinez, 2009). Ros (2008) also supports this idea, who considers that with these new tools, managing knowledge to generate value for the firm’s target must be the top priority.

The content generated on the brand pages is used by consumers to make better-informed purchase decisions or to obtain inspiration and new ideas (Muntinga, 2011). So, the content strategies used by firms on the social networks directly affect the engagement of the customer (Ashley and Tuten, 2015).

#### 4.1.2. Campaigns with advantages

According to McAlexander et al. (2002), people join together in brand communities with a view to obtaining economic benefits such as discounts or to participate in draws and competition; this idea is also supported by Tsai and Men (2013). Hansen and Lee (2013) state that the economic incentives greatly enhance the motivation of the users of social networks, who also relate to the transmission of the information in their own social network profiles. According to Huang et al. (2022), the benefits and special treatment obtained through social media positively influence loyalty.

The conclusions from the analysis conducted by Tsimonis and Dimitriadis (2014) also point in the same direction, and it can be deduced from their research that 50% of the activity that takes place on companies’ social networks involves communications about competitions.

Lee et al. (2019) show that when a brand offers incentives for sharing content, the number of times that this content is shared increases greatly, so this reveals the interest that the users of social networks show in the brand incentives.

It is also important to bear in mind that granting users incentives as rewards or giving them discounts could improve the users’ attention to publications, thereby promoting their participation and their brand loyalty. For example, the use of competitions could serve a way of encouraging engagement with the brand (Banerjee and Chua, 2019).

In the light of the above-mentioned studies, it is undoubtedly the case that the campaigns with advantages are one of the most important characteristics of the publications on social networks when it comes to assessing their ability to generate brand loyalty.

#### 4.1.3. Popular content among friends

The fact that consumers voluntarily share the contents of a brand on social networks is considered to be an indicator of the effectiveness of that brand’s publications (Alhabash and McAlister, 2015).

According to Fu et al. (2017), sharing the contents of brands and exchanging personal opinions about products and purchasing experiences gives a sense of achievement, self-expression and camaraderie. Users share content seeking psychological incentives, such as reputation and self-promotion or to help the firm to which the user is loyal or to help other consumers and users.

From the brands’ viewpoint, one of the most interesting characteristics of social networks is the viral nature of some of the publications, as opposed to the situation regarding traditional marketing, where there is only a one-way communication channel. In the case of social networks, not only is the channel two-way in nature, but it can also be multiplied exponentially through the virality of the messages published (Khan and Vong, 2014). According to Puspaningrum (2020) the central role of marketing on social networks as far as purchasing decisions are concerned, consists of being able to get customers to recommend to friends and relatives. Previous studies have concluded that brand can obtain major profits if they concentrate their efforts on reaching their followers’ friends, because it has been demonstrated that after the publication of content on social networks, approximately one third of the new openings of that brand’s website can come from the friends of the followers of the firm’s website on social networks (Quesenberry and Coolsen, 2019).

The content that is popular among friends is, according to the literature analyzed, the third most significant characteristic in making customers (Quesenberry and Coolsen, 2019). The impact of viral content is undeniable (Gruen et al., 2006) especially in digital environments.

It is also important to highlight that, according to Shukla et al. (2023), customers who engage with the brand and participate on social media, for instance, by sharing posts, are much more likely to exhibit loyalty to that brand.

These findings support the importance of popularity among friends, when it comes to the content created by a brand.

#### 4.1.4. Quality of the images

According to Martins et al. (2015) users like creative publications, with more images and less texts. In 2022 the most popular contents among the users of social networks in Spain were photos and videos (Castello-Martinez, 2009).

Some studies, such as Coursaris et al. (2016) and Oliveira and Figueira (2018) conclude that videos and visual content are the types of publications whose utilization is most widespread among the users of networks that are most successful.

The greater richness of the messages, conveyed by including photographs, videos and other graphic material, is associated with greater consumer participation, and thus greater involvement as regards having a sense of loyalty to the brand, as Niciporuc (2014) and Coursaris et al. (2016) point out.

According to Lee et al. (2019) consumers are more disposed to share the publications of brands when the content consists of entertaining videos, given that this will also be a way of offering entertainment to their friends.

Therefore, all the past studies would seem to indicate the importance of the fact that the images used in the publications are associated with the effectiveness of those publications and with the loyalty generated with the user.

#### 4.1.5. Social networks dimensions of the proposed model

A 4-dimension model is considered based upon the literature analyzed and making a review of the dimension of the characteristics of the publications on social networks with which to obtain loyal customers proposed by Erdogmus and Cicek (2012). The final dimension is removed from this model (Variety of platforms), because it does not apply to this study, which does not analyze the different platforms. However, a missing characteristic is detected to which the past literature does attach importance, namely the graphic quality of the publications, this explains why a decision has been taken to include this variable.

**FIGURE 10:**
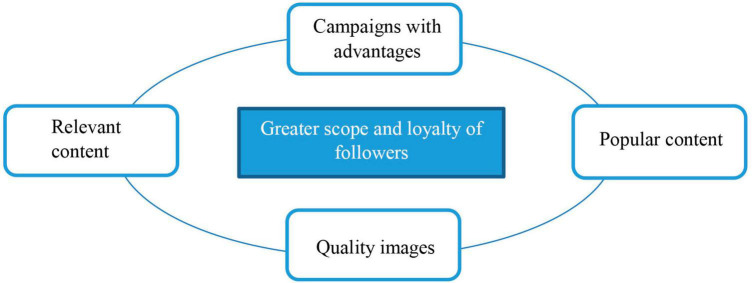
Significant characteristics of the publications from brands on social networks. Source: own research.

As a result, the following model shown in Figure 10 with 4 significant characteristics is obtained for the publications by brands on social networks regarding their capacity to attract the attention of the brand followers and their potential influence on loyalty.

### 4.2. Customer loyalty

Focusing on loyalty, the four-stage loyalty model by Oliver (1997) has been utilized in the literature to generate new models with that base. In contrast to other research work based on this model, this study proposes a simplified version featuring specific loyalty actions at each one of the stages, in order to offer a clearer and more practical view of the model. Therefore, each one of the loyalty phases is turned into an action or attitude of the customer that demonstrates his/her loyalty.

In their research, Moretta et al. (2019) establish the main customer loyalty variables, beginning with the emotional attachment to the brand. This attachment creates a bond of affection that makes consumers trust that brand (Gustafsson et al., 2005), who purchase it on repeated occasions and spread positive messages about it (recommendation) (Han et al., 2008). The result of such behavior ultimately leads to brand loyalty as (Iglesias et al., 2011) state. So, according to these authors and those listed below, when customers or consumers show these three attitudes or carry out those three actions described, they can be regarded as loyal customers.

#### 4.2.1. Repetition of purchase

All the authors being studied agree that brand loyalty contains a component of repeating purchase (Reichheld and Teal, 1996; Oliver, 1997).

According to the behavior current in customer loyalty, loyal customers are defined as those who carry on purchasing a product throughout time (Gupta and Zeithaml, 2006).

Loyal customers generate more income for their firms than occasional customers (Baumann et al., 2005). This is mainly due to the continued repetition of the purchase, amongst other factors, like being inclined to try new products, or to the cross-purchasing of products. Moreover, loyal customers are less sensitive to prices, given that on many occasions they think that higher prices can be interpreted as meaning better product quality or greater value for money, thereby bringing about an inverse relationship between loyalty and flexibility in demand, which once again leads to a greater frequency of purchase, as loyal customers tend to erect a certain barrier to keep the competition out (Labajo González and Tena Blázquez, 2009).

Nevertheless, in the analysis conducted by Reichheld and Teal (1996), they state that between 65 and 85% of consumers who switch brands, were satisfied with (but not loyal to) their previous brand. All of this shows that brand loyalty is much more than merely repurchasing, and being satisfied with a brand is not sufficient to guarantee that a person carries on purchasing it. According to Szymanski and Henard (2001) customer satisfaction accounts for less than 25% of the variations in intent to purchase.

These explanations show how repurchasing is not the same as being a loyal customer, and it also demonstrates how a satisfied customer does not necessarily become a loyal customer. Therefore, it is essential to incorporate other variables that account for loyalty in the model that is the fruit of this research.

#### 4.2.2. Trusting the brand

There is an attitudinal element to the complex nature of brand loyalty, which combines with the behavioral aspect (Bowen and Chen, 2001): trusting the brand plays a major role in that attitudinal aspect, and it is directly associated with brand loyalty (Christou, 2015).

Trust contains both a cognitive component and an emotional perception element. The former includes relative expectation regarding the consistency of the messages offered, credibility, competitiveness and the performance of a product or service (Delgado-Ballester et al., 2003) whereas the emotional perspective refers to the emotional expectations fulfilled and benevolence (Elliott and Yannopoulou, 2007).

According to Bruhn et al. (2014) a customer’s trust in a brand depends on the brand’s ability to meet its customers’ needs and interests. This enables the consumer to be certain that the product can fulfil the promised value and that the brand can prioritize the consumer’s interests. A trustworthy image helps the brand to maintain a long-term relationship of cooperation with its customers (Delgado-Ballester and Munuera-Alemán, 2005). So, trusting the brand is a factor that can establish an emotional tie between consumers and the firm, making them loyal.

According to Garbarino and Johnson (1999), satisfaction and trust in the brand, are important requisites to loyalty. Trust prompts customers to keep up a long-term relationship. Similarly, in this vein, Topor et al. (2022) assert that communications that engender customer satisfaction and customer trust also lead to customer loyalty. As emphasized in their study by Althuwaini (2022), social media posts have a direct impact on brand trust and customer loyalty. This notion is also supported by Attar et al. (2023).

Yet there are authors who claim that the quality of the product or service in themselves, which generate trust in the brand, are not sufficient to cement brand loyalty (Nyadzayo and Khajehzadeh, 2016). Once again, this leads to the conclusion that there is a need to incorporate into the model used in this research, a combination of explanatory variables for brand loyalty.

#### 4.2.3. Recommendation

The third pillar of loyalty to a brand that will be analyzed in this study could be considered an intermediate point between attitude and behavior, and this is recommending the brand.

According to Carroll and Ahuvia (2006), Gurău (2012), Yoo and Bai (2013), and Lee et al. (2019), consumers are loyal when they advise an acquaintance to use the brand. Going beyond this, the concept of proselytism emerges, which means that the consumer arouses other people’s interest in the product or service and somehow tempts them to try the brand to which they are loyal, in order to help it and affect its sale.

One of the benefits of brand loyalty, is that long-term customers or those that are stable owing to their loyalty, act as communication channels for the brand exchanging informal opinions with their network of friends and contacts, thus generating a beneficial word-of-mouth publicity for the brand (Wright and Sparks, 1999).

Kabiraj and Shanmugan (2011) that the most sought-after level of brand loyalty, “Sustainable Loyalty” is attained when apart from the aforementioned pillars, the customer is also responsible for a positive word-of-mouth interaction with other customers.

#### 4.2.4. Loyalty dimension with the proposed model

The study presented in this work, applies to loyalty exclusively in social networks, i.e., via publications on social networks access is gained to each one of the loyalty stages, it being considered as a premise, that a user who follows a brand, already has a high level of knowledge about that brand.

**FIGURE 11:**
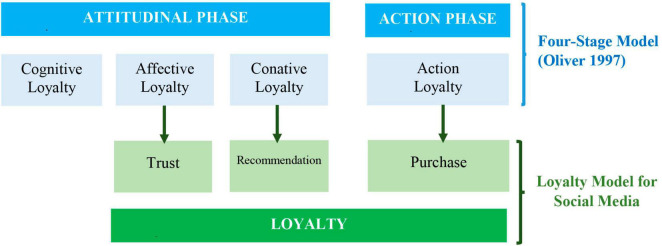
Proposed Loyalty model to be applied for social networks. Source: own research.

The proposed loyalty model for social networks can be regarded as Oliver’s Model evolved until it has the following structure, shown in Figure 11.

## 5. Final model

**FIGURE 12:**
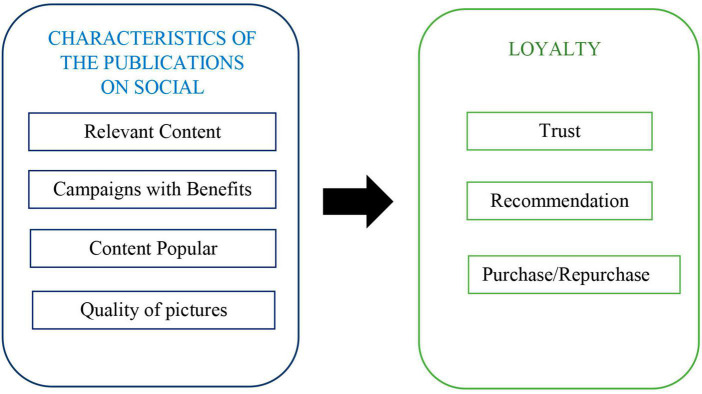
Proposed model to achieve loyalty via the social networks. Source: own research.

The final model that we arrive at with the union of the two models proposed above aims to consider a concept that can help companies to meet the need for them to have social media followers who will become loyal customers. It is a model that can be applied to any publication on a social network, regardless of the platforms used and the sector to which it belongs shown in Figure 12.

In compliance with the above statements, the proposed model finally has the following structure (Figure 12).

## 6. Discussion, conclusion, and future lines of research

### 6.1. Discussion

This work is comparable to previous studies in the sense that it explores both customer loyalty and communication on social networks, which have been subjects of examination in prior research. However, this study takes a unique approach by creating a new model that links the key variables of both concepts, thereby delving deeper into their practical application.

Numerous prior studies have solidified the connection between social media networks and customer loyalty. Aljuhmani et al. (2023) have underscored the model’s efficacy and its underlying constructs. This relationship has also been corroborated in earlier literature, exemplified by studies conducted by Zhou and Lu (2011), Lemon and Verhoef (2016), and Brun et al. (2017). Additionally, Tyrväinen et al. (2023) contend that characteristics of brand-generated social media content exert a direct influence on customer loyalty, aligning with the linkage proposed in the present study’s model.

The three-variable approach, encompassing customer loyalty [in terms of (re)purchase, trust, and recommendation, as indicated in this study’s findings], aligns with previous research, as referenced in section “4.2. Customer loyalty” of Erdogmus and Cicek (2012). These variables also resonate with Oliver’s Model (1997), upon which other studies like Tyrväinen et al. (2023) are based. In accordance with the model presented in this study, Nyadzayo and Khajehzadeh (2016) confirm that loyalty is a multivariable construct, and none of these three model variables can singularly imply brand loyalty unless combined with others.

However, it is important to note that while the current study builds upon previous research, it goes more in-depth than others in terms of practical application.

This article aligns with the conclusions of Hernández-Ortega et al. (2022), which assert that the features of brand-generated content on social media directly impact companies’ financial outcomes through customer loyalty. The proposed characteristics of social media content in this study’s model directly influence various aspects of loyalty, which, as Nyadzayo and Khajehzadeh (2016) contend, ultimately contribute to financial stability. Nevertheless, it is worth mentioning that no single author has unified the four variables in this study’s model into a single representation of the key features of social media.

In conclusion, this study delves deeper than previous literature into both the concepts of customer loyalty and the specific characteristics of social media posts, resulting in a more concrete, analytical, and applicable model for the practice of corporate marketing.

### 6.2. Conclusion and future lines of research

This research reviews different loyalty models based upon Oliver’s Model (1997), and the various marketing tools applied to the social networks proposed whose ultimate aim is to achieve loyalty, leading to a simplified model that can be applied to the business sector and the marketing sector. So companies whose aim is make their followers on social networks become loyal customers, can apply that model to create suitable publications that meet the real needs where making their followers loyal is concerned.

It is established that to achieve this goal, the four main characteristics for publications on social networks are: relevant content or content that generated added value, campaigns with advantages for the customer, content that is popular among friends and quality photographs, illustrations or animations (PQ).

The publications with these characteristics will give rise to types of publications that generate trust in the brand, which will lead to it being recommended causing the product or service to be acquired by customers and potential customers, and thus, loyal customers.

Based on the proposed model and the current situation in the use of social networks, a few lines of research are proposed below that could lead to new conclusions regarding this subject that may complete the proposals.

To test the proposed model, hypotheses of an attitudinal nature could be established as well as finding out the opinions of the social network users about the effectiveness of these types of publications and how they affect the different customer loyalty stages.

Furthermore, the reality of social network use (due mainly to the time they appeared and their access barriers) makes us think that it is highly likely that the effectiveness of this model could be affected by different sociodemographic variables that may affect the way users interact, not only with the brands but also with the social networks in general. That is why it would be of great interest to conduct an empirical study in which attitudinal differences are pinpointed based on such variables as age, generation, sex, education level, income bracket, geographical analysis: rural world vs. cities, etc.

The relationships between customers and their favorite brands are established in very different ways on the basis of the sectors to which the brand belongs, which is why an analysis by sectors would also shed light on this subject.

This model has been created on the basis of usability and on the social networks in general. However, each social network presents certain peculiarities: some enable users to include direct links in their publications, others do not. Some permit one type of image composition and others allow a very different type, etc. These peculiarities also give rise to differences in the publications that the brands can make when communicating with their customers. Therefore, by going more deeply into the matter, specific marketing action criteria could be established for loyalty in each one of the social networks.

In view of the great potential that implementing effective communication in social networks and brand loyalty have in terms of efficiency for companies and users, there is a great challenge to be met when continuing to discover the best ways to optimize relations between the two fields.

## Author contributions

ND: Conceptualization, Data curation, Formal analysis, Investigation, Software, Writing – original draft. MR: Funding acquisition, Methodology, Methodology, Resources, Supervision, Validation, Visualization, Writing – review and editing. CS: Funding acquisition, Project administration, Resources, Supervision, Validation, Visualization, Writing – review and editing.
